# Protective effect of autophagy on human retinal pigment epithelial cells against lipofuscin fluorophore A2E: implications for age-related macular degeneration

**DOI:** 10.1038/cddis.2015.330

**Published:** 2015-11-12

**Authors:** J Zhang, Y Bai, L Huang, Y Qi, Q Zhang, S Li, Y Wu, X Li

**Affiliations:** 1 Department of Ophthalmology, Peking University People's Hospital, Beijing 100044, China; 2 Key Laboratory of Vision Loss and Restoration, Ministry of Education, Beijing 100044, China; 3 Beijing Key Laboratory for the Diagnosis and Treatment of Retinal and Choroid Diseases, Beijing 100044, China; 4 Eye Institute of Xiamen University, Fujian Provincial Key Laboratory of Ophthalmology and Visual Science, Xiamen 361102, Fujian Province, China

## Abstract

Age-related macular degeneration (AMD) is the leading cause of central vision loss in the elderly. Degeneration of retinal pigment epithelial (RPE) cells is a crucial causative factor responsible for the onset and progression of AMD. A2E, a major component of toxic lipofuscin implicated in AMD, is deposited in RPE cells with age. However, the mechanism whereby A2E may contribute to the pathogenesis of AMD remains unclear. We demonstrated that A2E was a danger signal of RPE cells, which induced autophagy and decreased cell viability in a concentration- and time-dependent manner. Within 15 min after the treatment of RPE with 25 *μ*M A2E, the induction of autophagosome was detected by transmission electron microscopy. After continuous incubating RPE cells with A2E, intense punctate staining of LC3 and increased expression of LC3-II and Beclin-1 were identified. Meanwhile, the levels of intercellular adhesion molecule (ICAM), interleukin (IL)1*β*, IL2, IL-6, IL-8, IL-17A, IL-22, macrophage cationic peptide (MCP)-1, stromal cell-derived factor (SDF)-1, and vascular endothelial growth factor A (VEGFA) were elevated. The autophagic inhibitor 3-methyladenine (3-MA) and activator rapamycin were also used to verify the effect of autophagy on RPE cells against A2E. Our results revealed that 3-MA decreased the autophagosomes and LC3 puncta induced by A2E, increased inflammation-associated protein expression including ICAM, IL1*β*, IL2, IL-6, IL-8, IL-17A, IL-22, and SDF-1, and upregulated VEGFA expression. Whereas rapamycin augmented the A2E-mediated autophagy, attenuated protein expression of inflammation-associated and angiogenic factors, and blocked the Akt/mTOR pathway. Taken together, A2E induces autophagy in RPE cells at the early stage of incubation, and this autophagic response can be inhibited by 3-MA or augmented by rapamycin via the mTOR pathway. The enhancement of autophagy has a protective role in RPE cells against the adverse effects of A2E by reducing the secretion of inflammatory cytokines and VEGFA.

Age-related macular degeneration (AMD) is the leading cause of irreversible blindness among elderly people and is becoming a major public health issue.^1, 2, 3^ The pathological change in AMD is located in the macula, which is the central and posterior portion of the retina containing the retinal pigment epithelium (RPE) and photoreceptors. Central visual impairment caused by AMD results from the loss or damage of RPE cells and the photoreceptors.^4^ Currently, the etiology and pathogenesis of AMD is not fully understood and there is no effective treatment.^5, 6^ A chronic aberrant inflammatory response in RPE cells is considered to be one of the major factors contributing to the pathogenesis of AMD.^7, 8^

Lipofuscin is a complex aggregate of fluorescent material, formed in a variety of tissues but best studied in the eye.^9^ The buildup of lipofuscin in RPE cells has been identified as a byproduct of the visual cycle, and is derived from the ingestion of photoreceptor outer segments, which has been implicated in several retinal degenerations, including AMD.^10, 11^ As revealed by spectroscopic analyses, the bis-retinoid *N*-retinyl-*N*-retinylidene ethanolamine (A2E) is the first isolated, major fluorophore from RPE lipofuscin. Numerous *in vitro* and *in vivo* studies have found that toxicity effects associated with this compound, and A2E is involved in the pathological pathways of AMD, especially the inflammatory response.^12, 13^ Although several studies have suggested that A2E may induce cytokine production, activate inflammasomes or the complement system in RPE cells, and contribute to chronic inflammation in AMD,^14, 15, 16^ the exact mechanisms by which A2E exerts an effect on RPE cells remains unclear.

Autophagy is an evolutionarily conserved cellular housekeeping process that removes damaged organelles and protein aggregates that are unnecessary or dysfunctional to the cells by delivering cytoplasmic substrates to lysosomes for degeneration.^17^ In addition to turnover of cellular components, autophagy is involved in development, differentiation, and tissue remodeling in various organisms.^18^ The failure of autophagy in aged postmitotic cells, including RPE cells, can result in the accumulation of aggregation-prone proteins, cellular degeneration, and finally the induction of cell death.^19, 20^ Currently, a large amount of evidence indicates that autophagy is associated with RPE damage and AMD pathology.^21, 22, 23^ In RPE cells, the preservation of autophagic activity, together with functional lysosomal enzymes, is a prerequisite to prevent detrimental intracellular accumulation of damaged molecules.^21^ A well-functioning proteolytic machine guarantees that there is sufficient capacity to handle damaged proteins and organelles.^24^ In addition, Saadat KA *et al.*^25^ have shown that RPE cell death is induced in the presence of A2E and the autophagic inhibitor 3-methyladenine (3-MA). Nevertheless, whether the autophagic pathway has effects on A2E-induced cell damage through the production of chemokines and cytokines remains unclear. Furthermore, the relationship between A2E and autophagy and how this interaction influences RPE cells' inflammatory response requires further clarification.

Therefore, the protective effect of autophagy on human RPE cells against lipofuscin fluorophore A2E-induced cell death and the inflammatory response were studied in the present article. This work facilitates our understanding of the role of autophagy in the survival and death of RPE cells accumulating excess lipofuscin and provides a new strategy in the treatment of AMD.

## Results

### A2E inhibits RPE cell proliferation and induces the expression of inflammatory factors and an angiogenic cytokine

**Figure 1:**
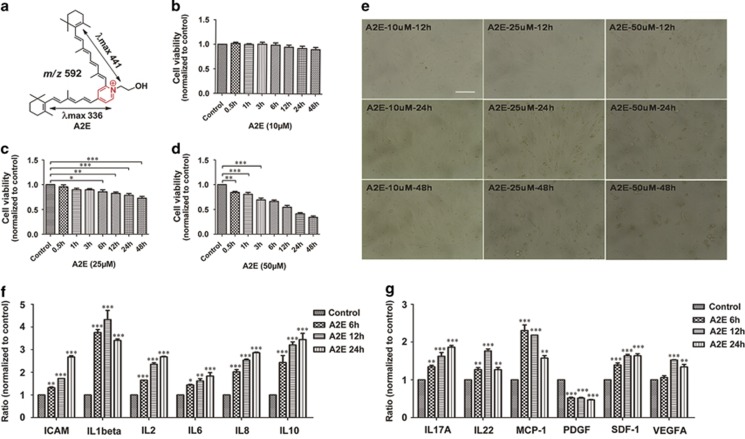
RPE cells exposed to A2E have reduced proliferation and increased cytokines expression. (**a**) Structure of A2E. Shown are UV-visible absorbance maxima (nm), mass-to-charge ratio (*m/z*), and electronic transition assignments (↔). (**b**−**d**) Cell viability 0.5, 1, 3, 6, 12, 24, or 48 h after introduction of A2E at concentrations of 10, 25, and 50 *μ*M, was probed by a CCK-8 assay. Data are presented as the means±S.E.M., *n*=6. *P<0.05, **P<0.01, ***P<0.001, ANOVA. A2E was dissolved in DMSO and cells exposed to only DMSO in culture medium served as a negative control. (**e**) Representative morphological images of RPE cells treated with 10, 25, and 50 *μ*M A2E for 12, 24, and 48 h. Scale bar: 100 *μ*m. (**f** and **g**) Multiplex cytokine assay was utilized to measure protein expression of inflammatory factors and angiogenic cytokines in RPE cells treated with 25 *μ*M A2E for 6 h, 12, and 24 h. Data are expressed as the means±S.E.M., *n*=3. *P<0.05, **P<0.01, ***P<0.001 *versus* control, ANOVA

RPE cells were incubated with varying concentrations (10, 25, and 50 *μ*M) of A2E (Figure 1a) for 0.5, 1, 3, 6, 12, 24, or 48 h. As shown in Figures 1b and e, 10 *μ*M A2E did not affect RPE cell viability at any time point. Cells treated with 25 *μ*M A2E displayed a time-dependent decrease in cell viability from 6 to 48 h (*P<0.05, **P<0.01, ***P<0.001) (Figures 1c and e). Treatment with 50 *μ*M A2E significantly decreased RPE cell viability from 30 min to 48 h (**P<0.01, ***P<0.001) and exhibited serious toxic effects on the RPE cells (Figures 1d and e). To explore the effect of A2E on RPE cells and its relationship to autophagy, we chose A2E at a concentration of 25 *μ*M for the following study.

The protein expression of 12 different human chemokines and cytokines was detected with a multiplex cytokine assay in RPE cells treated with 25 *μ*M A2E for 6 h, 12 h, and 24 h. Upon incubation with A2E, there was a significant upregulation of inflammation-associated chemokines and cytokines, including intercellular adhesion molecule (ICAM), interleukin (IL)-1*β*, IL-2, IL-6, IL-8, IL-10, IL-17A, IL-22, MCP-1, and SDF-1, in RPE cells at all time points (*P<0.05, **P<0.01, ***P<0.001, *versus* control, one-way analysis of variance) (Figures 1f and g). The angiogenic cytokine vascular endothelial growth factor A (VEGFA) was also significantly increased at 12 and 24 h (**P<0.01, ***P<0.001, *versus* control, one-way analysis of variance) (Figure 1g), whereas there was no increase in the level of platelet-derived growth factor at any time point (Figure 1g).

### A2E stimulates autophagy in RPE cells

**Figure 2:**
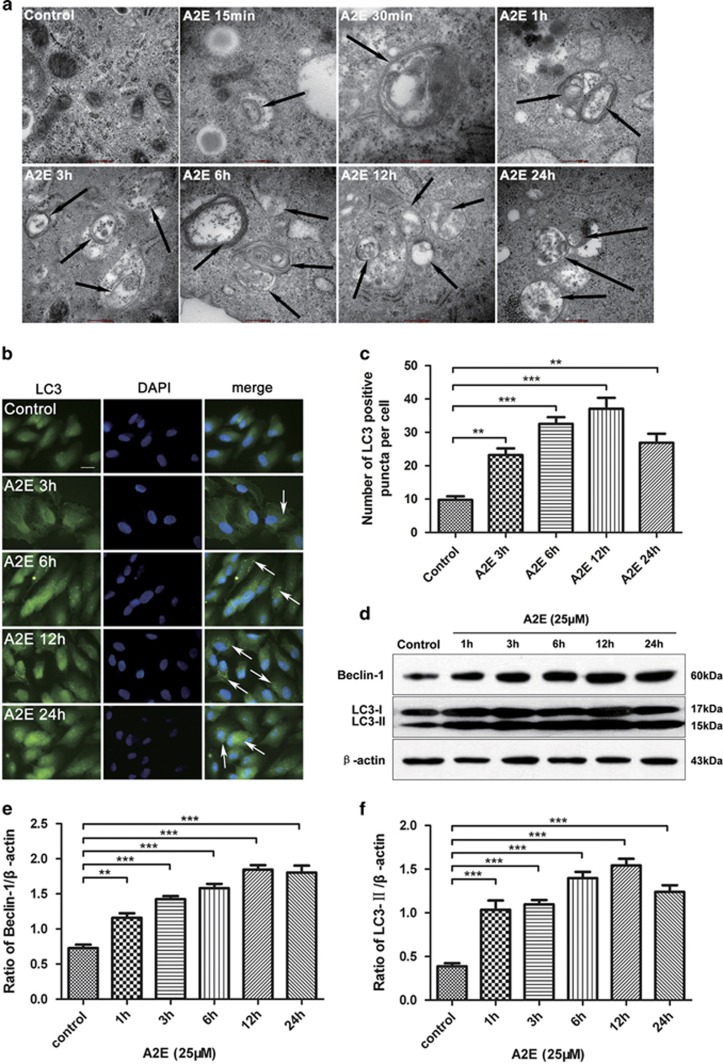
A2E stimulated autophagy in RPE cells. (**a**) Representative TEM photomicrographs of RPE cells exposed to 25 *μ*M A2E for 15 min, 30 min, 1, 3, 6, 12, and 24 h. Autophagic vacuoles are indicated by black arrows. Scale bar: 200 nm. (**b**) After being exposed to 25 *μ*M A2E for 3, 6, 12, and 24 h, representative images of RPE cells displaying LC3 puncta were immediately visualized by fluorescence microscopy. Nuclei were stained with DAPI. White arrows indicate the LC3-positive puncta. Scale bar: 20 *μ*m. (**c**) Quantification of the LC3-positive puncta per cell. Data are shown as the means±S.E.M., *n*=6. **P<0.01, ***P<0.001, ANOVA. (**d**) Expression levels of Beclin-1, LC3-I, and LC3-II protein in RPE cells at 1, 3, 6, 12, and 24 h after the introduction of 25 *μ*M A2E were quantified by western blot. *β*-Actin was used as the internal control. (**e** and **f**) Quantification of expression levels of Beclin-1 and LC3-II proteins. The data are presented as the means±S.E.M., *n*=3. **P<0.01, ***P<0.001, ANOVA

Few previous reports have described the details of A2E-induced autophagy. The formation of autophagosomes is one of the most important signs of autophagy and was observed in our study by transmission electron microscopy (TEM). The autophagy process begins with the formation of isolation membranes called phagophores. The latter then form double-membrane-bound autophagic vacuoles (autophagosomes) that contain oligomeric protein complexes and organelles. These autophagosomes fuse with lysosomes to form single-membrane-bound degradative vacuoles (autophagolysosomes).^18, 26^ In this study, in the presence of A2E (25 *μ*M), we found that phagophores were formed in RPE cells at 15 min; representative autophagosomes with double membranes were observed at 30 min, 1 h, and 3 h, and autophagolysosomes with a single membrane appeared at 6, 12, and 24 h (Figure 2a).

LC3 is a well-established marker of autophagosomes in mammalian cells. When autophagy occurs, punctate LC3 protein appears, and the soluble form of LC3 (LC3-I) is converted into the lipidated and autophagosome-associated form (LC3-II). In our study, after treatment with A2E, the localization of LC3 was clearly observed at 3, 6, 12, and 24 h (Figure 2b). The number of LC3-positive puncta reached a peak at 12 h and decreased at 24 h (Figure 2c). Western blot analysis indicated that the expression of both the Beclin-1 and LC3-II proteins increased at 1, 3, 6, and 12 h following A2E treatments and decreased at 24 h (Figures 2d and f). These findings demonstrated that autophagy was induced at an early stage in the RPE cells exposed to A2E.

### 3-MA inhibits A2E-induced autophagy in RPE cells

Our results described above revealed that A2E incubated with RPE cells induced autophagy, reaching a peak at 12 h (Figure 2), and significantly increased the levels of inflammatory factors and VEGFA (Figure 1). On the basis of previous reports that autophagy may have a cytoprotective role during various stresses,^27^ we used the autophagy-specific inhibitor 3-MA to elucidate the role of A2E-induced autophagy in RPE cells. The pre-treatment of RPE cells with 10 mM 3-MA for 1 h had no significant toxic effect.

**Figure 3:**
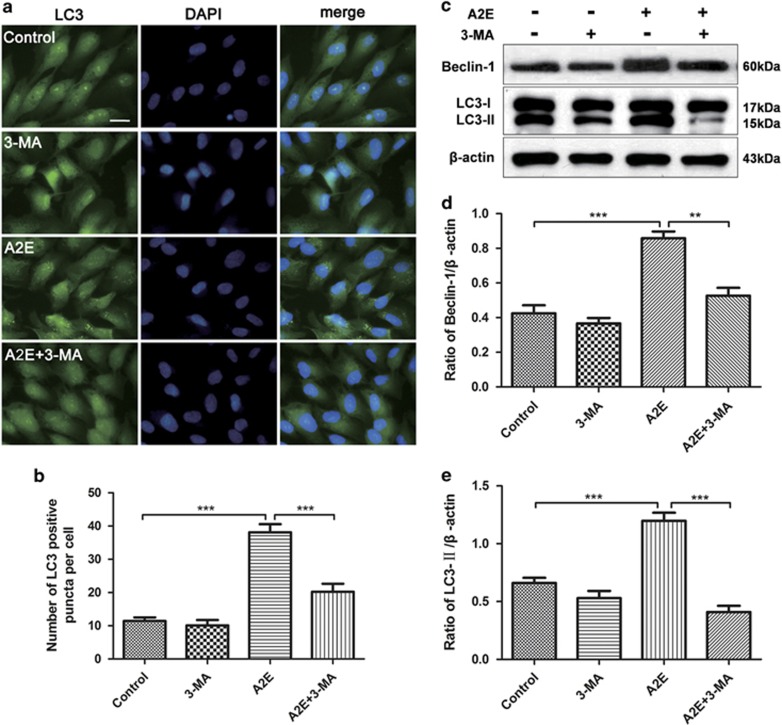
3-MA inhibited A2E-induced autophagy in RPE cells. (**a**) Fluorescence microscopy was used to detect the formation of LC3 puncta in RPE cells treated with 25 *μ*M A2E and/or 10 mM 3-MA for 12 h. Scale bar: 20 *μ*m. (**b**) Quantification of LC3-positive puncta per cell. Data are shown as the means±S.E.M., *n*=6. **P<0.01, ***P<0.001, ANOVA. (**c**) Western blot analysis for protein expression of Beclin-1, LC3-I, and LC3-II in RPE cells treated with 25 *μ*M A2E or A2E (25 *μ*M) combined with 10 mM 3-MA for 12 h. (**d** and **e**) Quantification of expression levels of Beclin-1 and LC3-II proteins. Data are presented as the means±S.E.M., *n*=3. **P<0.01, ***P<0.001, ANOVA

**Figure 4:**
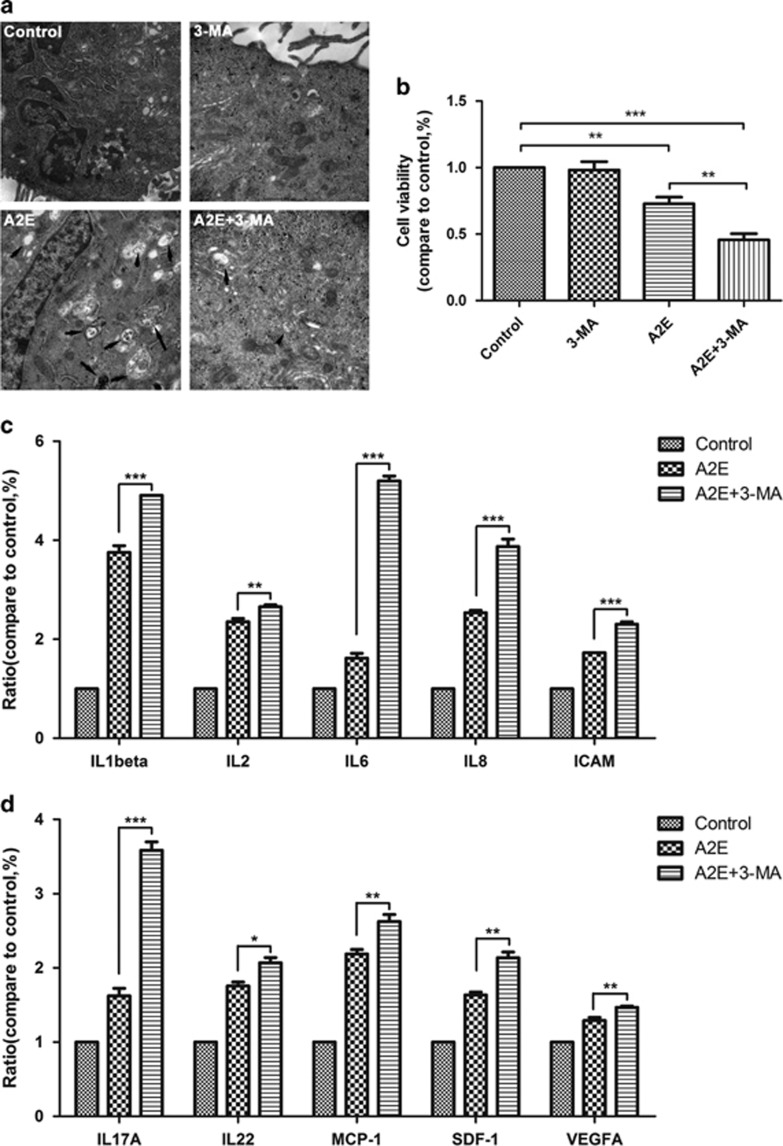
3-MA and A2E together exacerbated RPE cells death and stimulated cytokine production. Cells were exposed to 25 *μ*M A2E and/or 10 mM 3-MA for 12 h. (**a**) Typical TEM photomicrographs of RPE cells treated with A2E or A2E combined with 3-MA. Scale bar: 500 nm. (**b**) Cell viability was determined with the CCK-8 assay. Data are presented as the means±S.E.M., *n*=6. **P<0.01, ***P<0.001, ANOVA. (**c** and **d**) Protein expression of inflammatory factors and angiogenic cytokines were detected by multiplex cytokine assay in RPE cells treated with A2E or A2E combined with 3-MA. Data are presented as the means±S.E.M., *n*=3. *P<0.05, **P<0.01, ***P<0.001, ANOVA

After a 12-h incubation with A2E, LC3 puncta were clearly observed in the cells treated with A2E alone, but fewer LC3 puncta were observed in the cells treated with A2E combined with 3-MA (Figures 3a and b). We further monitored autophagy by assaying the expression levels of Beclin-1 and LC3-II. Cells treated with A2E in combination with 3-MA had reduced expression of LC3-II and Beclin-1 compared with cells treated with A2E alone (Figures 3c–e). Observation by TEM revealed that cells treated with A2E and 3-MA had fewer small autophagic vacuoles compared with the cells treated with A2E alone (Figure 4a). These results indicated that 3-MA inhibited A2E-induced autophagy in RPE cells.

### Inhibition of autophagy aggravates RPE cell death and increases the production of inflammatory factors and an angiogenic cytokine after A2E incubation

RPE cells were treated with 25 *μ*M A2E for 12 h with or without pre-treatment with 10 mM 3-MA for 1 h. Although Cell Counting Kit-8 (CCK-8) assays indicated that 3-MA alone had no significant effect on cell viability, 3-MA treatment enhanced the cytotoxic effect of A2E (Figure 4b). Protein expression of inflammatory factors and an angiogenic cytokine was detected using a multiplex cytokine assay in RPE cells treated with A2E or A2E combined with 3-MA. Compared with cells treated with A2E alone, the cells treated with A2E combined with 3-MA exhibited higher expression of inflammatory factors, including IL-1*β*, IL-2, IL-6, IL-8, ICAM, IL-17A, IL-22, MCP-1, and SDF-1, and the angiogenic cytokine VEGFA (Figures 4c and d).

### Rapamycin enhances A2E-induced autophagy in RPE cells

To further investigate whether the autophagy pathway might have a protective function, RPE cells were treated with A2E combined with rapamycin, which is an activator of autophagy.

**Figure 5:**
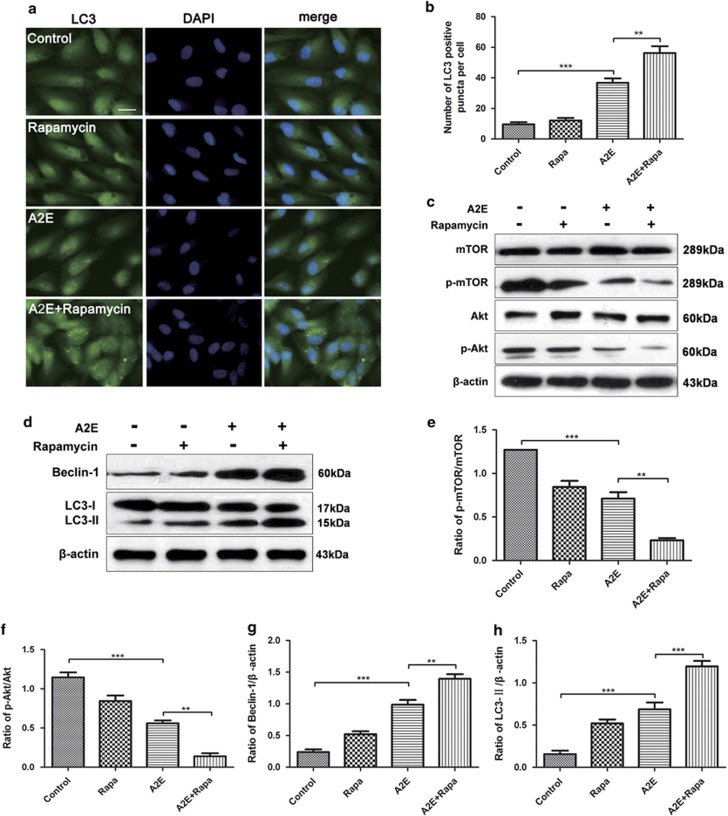
Rapamycin enhanced A2E-induced autophagy in RPE cells. (**a**) Fluorescence microscopy was used to monitor the formation of LC3 puncta in RPE cells treated with 25 *μ*M A2E and/or 100 nM rapamycin for 12 h. Scale bar: 20 *μ*m. (**b**) Quantification of LC3-positive puncta per cell. Data are shown as the means±S.E.M., *n*=6. **P<0.01, ***P<0.001, ANOVA. (**c**) Total levels of mTOR and Akt, together with phosphorylation levels of mTOR and Akt in RPE cells treated with A2E or A2E combined with rapamycin were determined with a western blot assay. (**d**) Expression of Beclin-1, LC3-I, and LC3-II protein in RPE cells treated with A2E or A2E combined with rapamycin were examined by western blot assay. (**e**−**h**) Quantification of expression levels of p-mTOR/mTOR, p-Akt/Akt, Beclin-1,and LC3-II proteins. Data are presented as the means±S.E.M., *n*=3. **P<0.01, ***P<0.001, ANOVA

**Figure 6:**
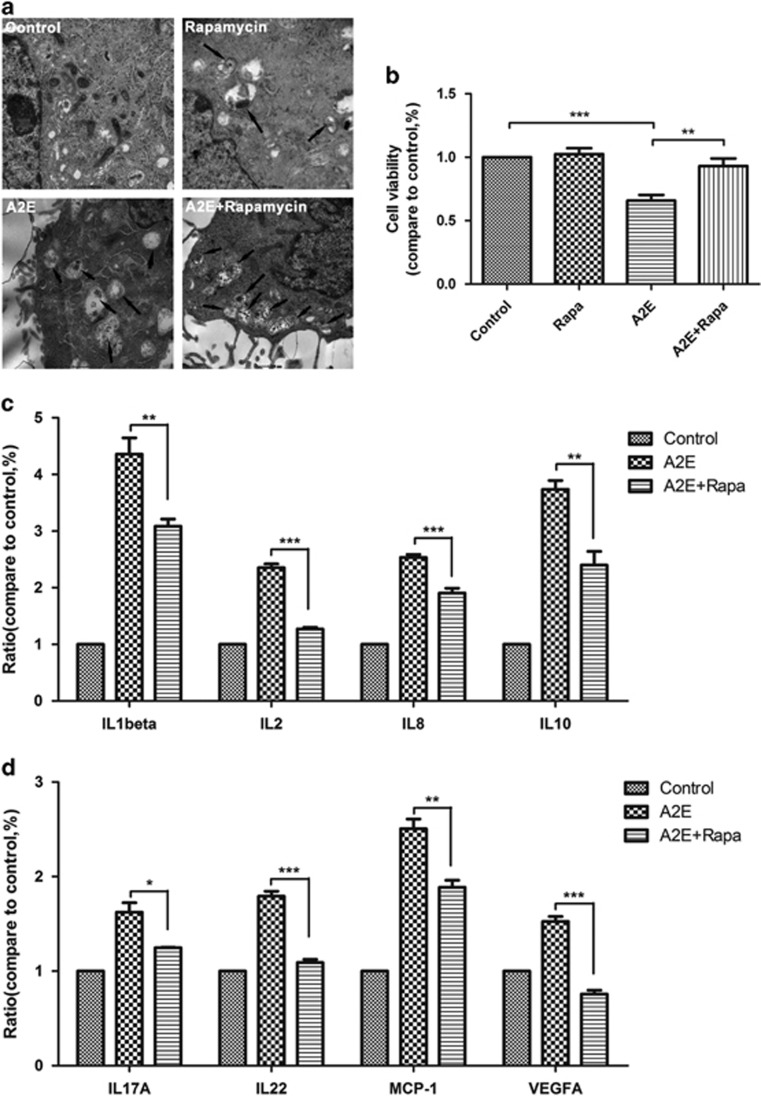
Rapamycin restored cell viability and cytokine production to normal levels in RPE cells incubated with A2E. RPE cells were treated with 25 *μ*M A2E and/or 100 nM rapamycin for 12 h. (**a**) Representative TEM photomicrographs of RPE cells treated with A2E or A2E combined with rapamycin. Scale bar: 500 nm. (**b**) Cell viability was probed by the CCK-8 assay. Data are presented as the means±S.E.M., *n*=6. **P<0.01, ***P<0.001, ANOVA. (**c** and **d**) Protein expression of inflammation factors and an angiogenic cytokine was detected via a multiplex cytokine assay in RPE cells treated with A2E or A2E combined with rapamycin. Data are presented as the means±S.E.M., *n*=3. *P<0.05, **P<0.01, ***P<0.001, ANOVA

Compared with cells treated with A2E alone, there were more LC3 puncta in the RPE cells treated with A2E combined with rapamycin as detected by immunofluorescence staining (Figures 5a and b). Western blot analysis revealed that rapamycin treatment increased the expression levels of Beclin-1 and LC3B-II in the A2E-treated cells (Figures 5d, g and h). Moreover, we observed more autophagic vacuoles after rapamycin pre-treatment of the A2E-treated RPE cells, as detected by TEM (Figure 6a).

To clarify the underlying mechanisms involved in these processes, the Akt/mTOR pathway was investigated. As shown in Figures 5c, e and f, A2E alone decreased the ratios of p-mTOR/mTOR and p-Akt/Akt in RPE cells compared with those found in the normal control. Our results additionally revealed that rapamycin pre-treatment further reduced the expression of p-mTOR and p-Akt in RPE cells treated with A2E. These data suggest that A2E probably activates autophagy through the Akt/mTOR signaling pathway and that rapamycin elevates autophagy in RPE cells treated with A2E via the inhibition of mTOR.

### Activation of autophagy ameliorates A2E-induced cell death and decreases the production of inflammatory factors and an angiogenic cytokine in RPE cells

RPE cells were treated with 25 *μ*M A2E for 12 h and/or pre-treated with 100 nM rapamycin for 1 h. Compared with the cells treated with A2E alone, cell viability was significantly enhanced by rapamycin combined with A2E, whereas rapamycin alone did not cause cell loss (Figure 6b). A multiplex cytokine assay revealed that rapamycin combined with A2E decreased the expression of inflammatory factors, including IL-1*β*, IL-2, IL-8, IL-10, IL-17A, IL-22, and MCP-1, compared with that of the cells treated with A2E alone (Figures 6c and d). The expression level of the angiogenic cytokine VEGFA was also clearly decreased (Figure 6d). Taken together, our observations demonstrate that autophagy has a protective function in RPE cells that have been treated with A2E for 12 h. The activation of autophagy by rapamycin rescues A2E-induced RPE cells and reduces the production of inflammatory factors and the angiogenic cytokine VEGFA.

## Discussion

Here, we demonstrated that (i) A2E, a major RPE lipofuscin fluorophore, is a damage signal in RPE cells, which will stimulate the secretion of several inflammatory factors and angiogenic cytokines; (ii) A2E incubation causes autophagy activation concentration- and time-dependently through the Akt/mTOR pathway; and (iii) the activation of autophagy has a protective effect on RPE cells against A2E by inhibiting the inflammatory response and reducing angiogenic cytokine VEGFA. These findings indicate that autophagy intervention will be valuable in preventing AMD development and progression.

The presence of drusen in the extracellular space between RPE cells and Bruch's membrane is a hallmark of AMD.^4^ Several components of drusen have been investigated, including lipofuscin, amyloid beta, complement, zinc, apolipoproteins, and others.^4, 28^ In all of these constituents, lipofuscin is one of the most important cellular deposits and forms as a result of the visual retinoid cycle.^9^ A major constituent of toxic lipofuscin is the di-retinal conjugate A2E, named because it is synthesized from all-*trans*-retinal and ethanolamine in a 2 : 1 ratio. A2E has a wedge-shaped structure consisting of a cationic pyridinium ring and two all-*trans*-retinal-derived side arms.^29, 30^ A2E is toxic to RPE cells *in vitro*, resulting in increased chronic oxidative stress and inflammation.^4, 31, 32, 33^ Most recently, Saadat KA *et al.*^25^ described that 10 *μ*M A2E did not induce RPE cell death. Indeed, we also found that low concentration of A2E did not lead to a loss of cell viability (Figure 1b, 10 *μ*M group). However, aberrant accumulation of A2E in the RPE is a progressive process. Several studies have reported that the A2E amount in the human RPE cells *in vivo* can reach a level of 60–130 ng per 10^5^ cells^34^ or 830 pmol per eye,^35^ which is comparable with the bis-retinoid level in the RPE cells incubated *in vitro* for several hours with 15–30 *μ*M A2E.^36, 37^ The level at which A2E is presented in our experiments is 25 *μ*M, and is identical with what is expected *in vivo*. As expected, we observed that A2E becomes toxic at higher concentrations (Figure 1a, 25 and 50 *μ*M groups), and a 6-h incubation with 25 *μ*M A2E resulted in RPE cell death (Figure 1c, 25 *μ*M group) and the upregulation of inflammatory cytokines and an angiogenic factor (Figures 1f and g). The concentration- and time-dependent response is similar to clinical findings demonstrating that lipofuscin deposition occurs over a lifespan.^38^ Therefore, the search for early responses of RPE cells to toxic substances will be very valuable in clinical interventions.

Autophagy has an important role in maintaining cellular homeostasis by removing dysfunctional organelles and proteins.^39^ Deficient autophagy results in damaged cell viability because of several processes, including reactive oxygen species generation, lysosomal leakage, and impaired mitophagy.^40^ Autophagy-related proteins have recently been found to be strongly expressed in the retina.^21, 27^ Furthermore, there is strong evidence that mitochondrial damage and defective autophagy are features of the aging retina, and these processes are further exacerbated in AMD.^21, 41^ In addition, several studies have shown that autophagy is associated with RPE damage; for example, lipid peroxidation products reduce RPE autophagy and increase lipofuscin accumulation.^42^ Although Saadat *et al.*^25^ reported that A2E augmented RPE autophagy after a 12-h incubation, we further explored the response of A2E to RPE cells with regard to autophagy in the present study. We found that after only 15 min of incubation with A2E (25 *μ*M), RPE cells initiated the process of autophagy on the basis of observations via TEM of double-membrane-bound autophagic vacuoles (autophagosomes). The latter formed single-membrane-bound degradative vacuoles (autophagolysosomes) in the RPE cells after 6 h (Figure 2a). In addition, the course of autophagy was also confirmed by the buildup of a punctate pattern of cytosolic LC3 and the upregulation of LC3-II and Beclin-1 (Figures 2b and f), which are important markers of autophagosome formation.^43, 44^ Meanwhile, A2E-induced autophagy is a time- and concentration-dependent dynamic process. As shown in Supplementary Figure 1, autophagy was enhanced significantly as the incubation occurred in the 10 *μ*M A2E treatment group (Supplementary Figure 1, 10 *μ*M treatment group). However, in the 25 and 50 *μ*M treatment groups, A2E-induced autophagy was upregulated first, and downregulated thereafter (Supplementary Figure 1, 25 and 50 *μ*M treatment groups). Although A2E at higher concentrations is clearly directly toxic to RPE cells, it also induces by itself autophagy, which subsequently has a protective effect. Future studies will be needed to determine what circumstances influence the balance between these two effects.

To determine the significance of autophagy in A2E-treated RPE cells, the autophagy inhibitor 3-MA and activator rapamycin were used.^26^ Our data indicated that 3-MA decreased A2E-induced LC3 puncta and the expression of LC3-II and Beclin-1, but increased the rate of A2E-induced cell death (Figure 4). Furthermore, a multiplex cytokine assay revealed that 3-MA increased the A2E-induced expression of inflammatory factors and an angiogenic cytokine (IL-1*β*, IL-2, IL-6, IL-8, ICAM, IL-17A, IL-22, MCP-1, SDF-1, and VEGFA) in RPE cells (Figure 5). By contrast, rapamycin manifested the opposite effect of 3-MA; rapamycin rescued RPE cells from the cell damage induced by A2E and inhibited the expression of several cytokines that were normally increased by A2E, including IL-1*β*, IL-2, IL-8, IL-10, IL-17A, IL-22, MCP-1, and VEGFA (Figure 5 and Figure 6). Previous evidence indicated that inflammation-related cytokines have important roles in dry AMD development, and VEGFA is the key regulator of neovascular AMD formation.^28^ As depicted in Figure 2, autophagy resulting from 25*μ*M A2E increased with the accumulation of incubation time up to 12 h, but significantly decreased at 24 h, probably because autophagic dysfunction occurred and thereby inflicted further irreversible loss of RPE cells after a 12 h incubation. Our findings in this work suggest that the upregulation or activation of autophagy may reduce cell damage caused by the excess deposition of A2E and may provide a new target for AMD treatment. However, our present research lacks the support of *in vivo* experiments, and further studies will be required in the future.

The Akt/mTOR pathway is a classic intracellular signaling pathway that is known to be involved in autophagy.^45, 46^ mTOR, the mammalian target of rapamycin, is an important protein kinase that regulates cellular functions such as cell growth, protein synthesis, and transcription.^47^ mTOR inhibits the initiation of phagophore formation and blocks the earliest step of autophagy, thereby leading to decreased formation of autophagosomes and the expression of LC3B-II.^48, 49^ Early reports have confirmed that mTOR regulates the detrimental dedifferentiation and hypertrophy of RPE cells that have been exposed to oxidative stress, whereas treatment with rapamycin can prevent these effects and preserve photoreceptor function.^50^ Rapamycin has also been observed to alleviate choroidal neovascularization by inhibiting the function of VEGFA.^51^ In this publication, we revealed that A2E decreased the expression of p-Akt and p-mTOR and that rapamycin further inhibits the Akt/mTOR signaling pathway, thereby elevating the levels of autophagy in A2E-treated RPE cells. The results showed that the levels of phosphorylated proteins involved in the Akt/mTOR pathway were significantly decreased, and the autophagy induced by A2E was enhanced by rapamycin in the RPE cells.

Collectively, these findings indicate that autophagy activation protects RPE cells from A2E-induced damage. Our study provides a more complete understanding of the relationship of A2E-induced autophagy in RPE degeneration and could open avenues toward target therapies for AMD. Although some researchers have suggested that the regulation of autophagy may be a potential therapeutic strategy for AMD prevention or therapy, targeting a process as complex as autophagy is likely to be more difficult than previously thought. Further experiments will be fundamentally important in providing the guidance required to make the targeting of autophagy clinically useful.

## Materials and Methods

### Cell culture

Human RPE cells (ARPE-19 cell line) were obtained from the American Type Culture Collection (CRL-2302, Manassas, VA, USA) and were cultured in Dulbecco's modified Eagle's medium/F12 human amniotic membrane nutrient mixture containing penicillin and streptomycin (DMEM/F12; Sigma-Aldrich, St. Louis, MO, USA) with 10% fetal bovine serum (Invitrogen-Gibco, Grand Island, NY, USA) at 37 °C in a humidified atmosphere of 5% CO_2_. The cells were treated according to the experiments being performed and were not used beyond passage 35.

### A2E synthesis and cell treatment

A2E was generated using a one-step biosynthetic reaction of all-trans-retinal (two equivalents) and ethanolamine (one equivalent) in ethanol as previously described.^35^ For the isolation and preparation of the fluorophore, the reaction mixture was concentrated using a rotary evaporator and subjected to reverse-phase HPLC. A preparative scale YMC-Pack ODS-AC18 column (5 *μ*m, 10 mm × 250 mm; YMC Co., Ltd., Kyoto, Japan) was first used with an acetonitrile and water gradient containing 0.1% trifluoroacetic acid (83–90% acetonitrile, 0–5 min; 90–95% acetonitrile, 5–15 min; 95–100% acetonitrile, 15–30 min; 100% acetonitrile, 30–50 min; flow rate, 2 ml/min). Further purification was performed on an analytical-scale Atlantis dC18 column (3 *μ*m, 4.6 mm × 150 mm; Waters Corp., Milford, MA, USA) with a gradient of acetonitrile in water with 0.1% trifluoroacetic acid: 85–100% acetonitrile (0–15 min, 0.8 ml/min), 100% acetonitrile (15–20 min, 0.8–1.2 ml/min) and 100% acetonitrile (20–40 min, 1.2 ml/min). The photodiode array detector was set at 430 nm to monitor the eluent. HPLC-grade A2E was obtained for subsequent studies (Figure 1a).

For uptake into cultured human RPE cells, A2E was delivered in culture medium at concentrations of 10, 25, and 50 *μ*M, and confluent cultures of the cells were incubated with A2E or DMSO (1/10 000 control) for the indicated times. For the treatment group, the cells were pre-treated with the autophagic inhibitor 3-MA (10 mM, M9281, Sigma-Aldrich) or the autophagic activator rapamycin (100 nM, R8781, Sigma-Aldrich) for 1 h before the A2E incubation.

### Cell proliferation assay

Human RPE cells were plated in 96-well plates at a density of 5000 cells per well in 100 *μ*l of complete culture medium and allowed to adhere for 24 h. Each experimental group was treated in at least six wells. At each time point, the supernatant was removed, and 100 *μ*l of culture medium containing 10 *μ*l of Cell Counting Kit-8 reagent (CCK-8; Dojindo, Shanghai, China) was added to each well; the incubation was continued for 2 h at 37 °C. The absorbance was then recorded with an ELISA microplate reader (Finstruments Multiskan Model 347; MTX Lab Systems, Inc., Vienna, VA, USA) at 450 nm. All the experiments were independently repeated three times.

### Transmission electron microscopy (TEM)

Cells were treated with 25 *μ*M A2E for 15 min, 30 min, 1, 3, 6, 12, and 24 h. Cell culture samples were prefixed with 2.5% glutaraldehyde in 0.1 M phosphate buffer pH 7.4 for 2 h at room temperature. After a 15 min wash in 0.1 M phosphate buffer, the samples were post-fixed in 1% osmium tetroxide and 0.1 M phosphate buffer for 1 h and again washed with phosphate buffer for 15 min prior to standard ethanol dehydration. Subsequently, the samples were infiltrated and embedded in LX-112 resin (Ladd Research, Williston, VT, USA). Polymerization was conducted out at 37 °C for 24 h and at 60 °C for 48 h. Sections were cut with a Leica Ultra microtome UCT (Wetzlar, Germany) and imaged with a FEI Tecnai Spirit transmission electron microscope (TEM, FEI, Hillsboro, OR, USA).

### Western blot analysis

RPE cells were harvested, lysed in RIPA buffer (1% Nonidet P-40, 0.5% sodium deoxycholate, 0.1% SDS in PBS), and centrifuged at 15 000 r.p.m. for 15 min at 4 °C. Equal amounts of proteins were separated by 10% SDS-PAGE and transferred electrophoretically to polyvinylidenedifluoride membranes (Amersham, Little Chalfont, UK). Membranes were blocked with 5% non-fat milk for 1 h and incubated overnight at 4 °C with the following primary antibodies: rabbit anti-LC3 (1 : 1000, ab48394, Abcam, San Francisco, CA, USA); rabbit anti-Beclin1 (1 : 1000, ab16998, Abcam); rabbit anti-mTOR (1 : 1000, T2949, Sigma-Aldrich); rabbit anti-phospho-mTOR (anti-p-mTOR, 1 : 1000, #2971, Cell Signaling Technology, Danvers, MA, USA); rabbit anti-Akt (1 : 1000, #4685, Cell Signaling Technology); rabbit anti-phospho-Akt (anti-p-AKT, 1 : 1000, #4060, Cell Signaling Technology); and rabbit anti-*β*-actin (1 : 1000, #4970, Cell Signaling Technology). The membranes were then incubated with goat anti-rabbit horseradish peroxidase-conjugated secondary antibody (1 : 3000, #7074, Cell Signaling Technology) for 1 h at room temperature. The density of each band was analyzed with Image J software. Each experiment was repeated three times.

### Immunofluorescence staining

Cells were fixed with 4% paraformaldehyde in PBS for 10 min at room temperature. To determine the localization of LC3, permeabilization was achieved with 0.2% Triton X-100 in PBS for 15 min at 4 °C. Samples were blocked with 3% fat-free dry milk in 0.05% Tween/TBS for 30 min at room temperature. Anti-rabbit LC3 antibody (1 : 100, ab48394, Abcam) was diluted in 0.05% Tween/TBS and incubated with the samples at 4 °C overnight. After three 5-min washes with PBS, the samples were incubated for 1 h at room temperature with secondary anti-rabbit (Alexa Fluor 488; Invitrogen, Carlsbad, CA, USA) antibody diluted 1 : 1000 in 2% BSA/PBS. The cell nuclei were counterstained with DAPI (1 mg/ml). After a final wash, the slides were mounted and analyzed with a fluorescence microscope.

### Multiplex immunoassay

A Procarta cytokine profiling kit (Panomics, Santa Clara, CA, USA) was used according to the manufacturer's instructions to simultaneously detect 12 different human chemokines and cytokines in cell culture supernatants. Briefly, 50 *μ*l of antibody beads was added to each well of the filter plate and washed with wash buffer. A volume of 50 *μ*l of cell culture supernatant was then added to each well, incubated for at least 1 h at room temperature, and washed with wash buffer. Afterward, 25 *μ*l per well of the Detection Antibody solution was added, and the filter plate was shaken at 500 r.p.m. for 30 min at room temperature. After adding streptavidin-PE, the signals were detected with a Luminex 200 instrument (Bio-Rad, Hercules, CA, USA). The chemokines and cytokines included in the present study were inflammation-associated factors and angiogenic cytokines (ICAM, IL-1*β*, IL-2, IL-6, IL-8, IL-10, IL-17A, IL-22, MCP-1, platelet-derived growth factor, stromal cell-derived factor (SDF)-1, and VEGFA). The results were based on three independently repeated experiments (Supplementary Table 1).

### Statistical analysis

Data analysis was performed using the following statistical software programs: Prism 5 (GraphPad Software Inc., San Diego, CA, USA) and SPSS (SPSS, version 17.0; SPSS Science, Chicago, IL, USA). All the data are presented as the means±S.E.M., and the normality of the distribution was assessed. Individual group means were compared using Student's unpaired *t*-test, and data sets were examined by a one-way analysis of variance followed by a *post hoc* Dunnett's *t*-test. For group comparisons, mixed linear models were used. Differences with a *P*-value<0.05 were considered statistically significant.

## Abbreviations

AMD: Age-related macular degeneration
RPE: retinal pigment epithelial
ICAM: intercellular adhesion molecule
IL: interleukin
MCP: macrophage cationic peptide
SDF: stromal cell-derived factor
VEGFA: vascular endothelial growth factor A
3-MA: 3-methyladenine
VEGF: vascular endothelial growth factor
CCK-8: Cell Counting Kit-8
TEM: transmission electron microscopy
