# Supplementary material for: Protective effect of autophagy on human retinal pigment epithelial cells against lipofuscin fluorophore A2E: implications for age-related macular degeneration
Source: Cell Death Dis. 2015 Nov 12;6(11):e1972–. doi: 10.1038/cddis.2015.330 (PMC4670934; doi:10.1038/cddis.2015.330)
Supplement: Supplementary Information [file cddis2015330x1.docx]

Supplementary Table 1.The concentration of chemokines and cytokines in the control group.

| Chemokines and Cytokines | $\bar{x}$±s.e.m (pg/ml) |
| --- | --- |
| ICAM  IL1beta  IL2  IL6  IL8  IL10  IL17A  IL22  MCP-1  PDGF  SDF-1  VEGFA | 25.515±2.444  0.308±0.014  4.907±0.738  310.050±22.712  818.099±54.105  0.285±0.080  5.784±1.844  125.958±11.548  49.233±8.513  12.164±0.588  530.792±26.150  1119.937±29.456 |

ICAM: intercellular cell adhesion molecule; IL: interleukin; MCP: monocyte chemotactic protein; PDGF: platelet-derived growth factor; SDF: stromal cell-derived factor; VEGF: vascular endothelial growth factor.

**Supplementary Figure 1: A2E stimulated autophagy in RPE cells in a time-dependent and concentration-dependent manner.**

**
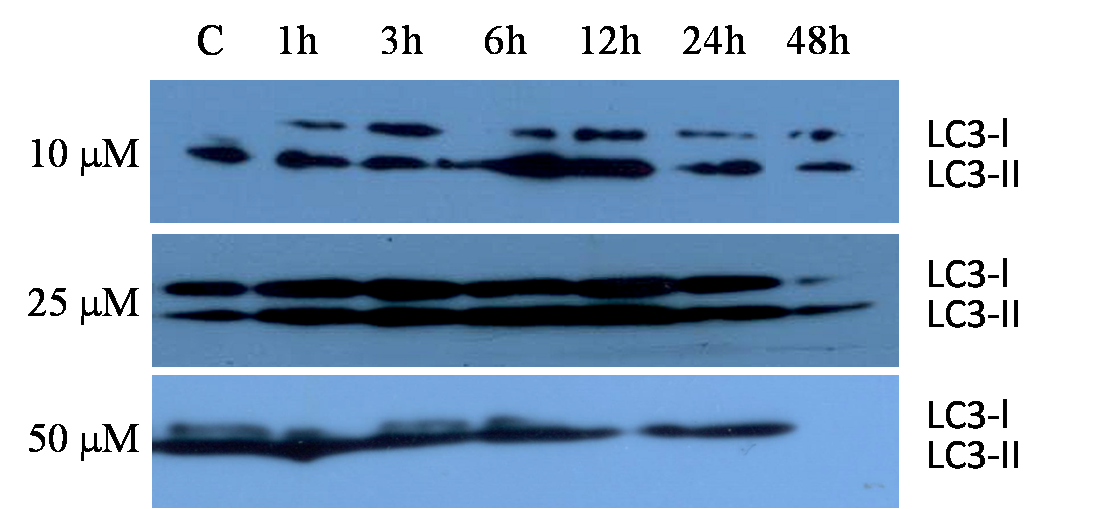
**

In the 10μM A2E treatment group, autophagy was enhanced significantly as the incubation time lasted. In the 25μM and 50μM treatment groups, A2E induced autophagy was first up-regulated first, and decreased thereafter.
